# Supplementary material for: Ancient hybridization fuels rapid cichlid fish adaptive radiations
Source: Nat Commun. 2017 Feb 10;8:14363. doi: 10.1038/ncomms14363 (PMC5309898; doi:10.1038/ncomms14363)
Supplement: Supplementary Information — Supplementary Figures, Supplementary Tables, Supplementary Discussion, Supplementary Methods and Supplementary References [file ncomms14363-s1.pdf]

Supplementary Methods

Mitochondrial chronograms

Dated phylogenies were reconstructed based on mitochondrial D-loop[69] and ND2[68] sequences using BEAST v. 2.3.0[75]. Three partitions were applied, one for D-loop, one for the first and second codon positions of ND2, and one for the third codon positions of ND2. We used the best-fit substitution models selected with the AIC criterion in jModelTest 2.1.7[76] which were GTR+G +I for D-loop and ND2 first and second codon positions and GTR+G for the third codon positions of ND2. An uncorrelated relaxed clock model with log normal distribution was used in runs of 20 million generations (after 2 million generations burn-in) with a calibrated Yule prior for the tree model. To follow the Yule model assumption of constant birth rates for all branches leading to symmetric trees and to avoid node sampling bias and mutational saturation, we optimized the tree symmetry and evenness of node sampling by using only a subset of taxa for every clade and excluded the most divergent outgroup taxa. We applied four different sets of calibration points for the Lake Malawi radiation age and the age of the "modern haplochromines" (sensu Salzburger *et al.*, 2005) including calibrations based on i) lacustrine paleoenvironment[78], ii) non-cichlid fossils[79], iii) cichlid fossils[37], and iv) the Gondwanan breakup[37,80] (Supplementary Table 3). The prior estimates for the Lake Malawi radiation age and the "modern haplochromines" ranged from 0.57 to 4 and from 3.4 to over 8.5 million years, respectively (Supplementary Table 3). Chain stationarity and run parameter convergence (ESS>200) were confirmed with Tracer 1.6[81]. Maximum clade credibility trees with 95% highest posterior density (HPD) intervals from TreeAnnotator 2.3.0[81] were visualized in FigTree (http://tree.bio.ed.ac.uk/software/figtree).

Estimating very recent divergence times with relaxed molecular clock approaches is challenging due to non-linearity of the clock rate in the very recent time range (reviewed in Ho, *et al.*[82]). Evidence for increasing clock rates towards the present has been accumulating in a wide range of taxa, where close to the present the molecular clock approaches the mutation rate, but moving backwards in time this rate declines exponentially, finally approximating the much lower substitution rate[82]. Generally, substitution

rates are only reached after about one million years[82,83] a finding in agreement with cichlid mitochondrial markers[37]. No molecular clock inference approaches incorporating this recent time dependency yet exist. Thus the calibration approach we have taken is useful to infer split times for divergence events more than one million years ago, but will result in an overestimate of recent divergence times. However, given that the node we aim to date (Congo-Nilotic divergence time) is in the time range where the molecular clock is constant and its age is similar to the age of the calibration nodes, we believe that this problem should not affect the estimate of the divergence time between the Congolese and the Upper Nile lineage. To get an indication of relative ages in the very recent part of the tree, we have added a clade endemic to a lake for which the geological age is well-constrained (Lake Nshere, 50,000 years)[37,84].

To compare the splitting time between cichlid lineages with the major relevant geological events, we reconstructed paleogeographic maps at different time points based on previously published data and reviews[85-94].

Supplementary Discussion

The Egypt clade and *Haplochromis* sp. "Nyangara"

The Egypt clade consists of individuals sampled from several sites in the Egyptian Nile river delta system. In the mitochondrial phylogeny, all Egypt individuals form a clade nested within the Edward/Kivu/Albert subclade of the Lake Victoria Region Superflock, and in the nuclear phylogeny, these individuals form a well-supported clade that is sister to the entire Lake Victoria Region superflock (Fig. 1, Supplementary Fig. 1). In ABBA-BABA tests, the Egypt clade, like all groups within the LVRS, shows elevated D statistics if tested for Upper Nile taxa gene flow (Supplementary Table 2). In fact, the Upper Nile ancestry proportion is even higher in the Egypt clade (α=0.37, Supplementary Table 4). A scenario consistent with these findings would be that the Egypt clade is derived from the LVRS, and after spreading down the Nile, received secondary gene flow from Upper Nile taxa or a closely related lineage. This is not unlikely given its biogeographic distribution in the Nile river delta system.

The *Haplochromis* sp. "Nyangara" individual was collected in the Rusizi River delta. The Rusizi River is the outflow of Lake Kivu into Lake Tanganyika, and hence the Congolese watershed, which formed after the uplift of the Virunga mountains (~11,000-25,000 years ago) in the North of Lake Kivu blocked the former Lake Kivu outlet into the Nile watershed[1,2]. In the nuclear phylogeny, *H.* sp. "Nyangara" is the sister taxon to the LVRS including the Egypt clade, whereas in the mitochondrial tree, it falls within the Edward/Kivu/Albert subclade of the LVRS/Egypt clade. D statistics show that *H*. sp. "Nyangara" has some Upper Nile lineage ancestry contribution (D=0.12, Supplementary Table 2) but to a lesser extent than LVRS (6% as estimated with the F4-ratio test, Supplementary Table 4). Morphologically, it looks like a generalized member of the LVRS. As is also the case for the Egypt clade, the mitochondrial haplotype of *H.* sp. "Nyangara" suggests that it shares a common ancestor with the western rift lake subclade (Edward/Kivu/Albert) of the LVRS. The smaller Upper Nile ancestry proportion indicates that *H.* sp. "Nyangara" may have received additional alleles from *A. stappersi* or a related Congolese species after it entered the Lake Tanganyika/Congo drainage.

The cyto-nuclear discordance of "*Haplochromis*" *gracilior*

Thoracochromis pharyngalis and *"Haplochromis" gracilior* are sister species in the nuclear genome and they behave identically in the ABBA-BABA tests showing strong signals of having contributed genes to the admixed ancestry of all tested Lake Victoria Region radiations. Lake Kivu used to drain to the North, feeding Lake Edward and the upper Nile until the end of the Pleistocene, and only underwent flow reversal to feed into Lake Tanganyika and the Congo after major volcanic activity had blocked the northern outlet[2]. So a sister species relationship between *T. pharyngalis* and *H. gracilior* makes sense biogeographically. However, the mitochondrial haplotype of *H. gracilior* is distinct and deeply divergent both from *T. pharyngalis* and the close relatives of the mitochondrial lineage of the latter, the taxa found in the Eastern Tanzanian Rivers. This finding suggests that *H. gracilior* and/or *T. pharyngalis* have a history of hybridization with mitochondrial capture from a third distinct lineage that has generated this cyto-nuclear discordance. There is no other species known that has a mitochondrial haplotype closely related to the one found in *H. gracilior* making further investigations of its evolutionary history difficult, though not impossible with whole-genome sequences[3]. Given that *H. gracilior* behaves identically to *T. pharyngalis* in all tests for introgression into the ancestor of all Lake Victoria Region radiations, the history of hybridization in *H. gracilior* does not affect the conclusions about the Congo-Nilotic hybridization event at the base of the LVRS.

***LWS* opsin haplotypes**

The haplotypes found in the Congolese and Upper Nile lineage taxa, respectively, have basal positions in the two major *LWS* opsin haplotype clades found in the LVRS, often referred to as classes I and II[4] (Fig. 4, Supplementary Fig. 7). These haplotype classes differ in many amino acid substitutions, some of which have known effects on peak wavelength sensitivity. Our finding of ancestry informative intronic SNPs and a 6 bp long indel (Supplementary Data 3) rules out the possibility that strong parallel selection and sequence

convergence could explain the similarity between the two major haplotype classes in the LVRS and the Upper Nile and Congolese lineage haplotypes, respectively.

To study the phylogenetic history of the two major *LWS* opsin haplotype classes I and II[4] in the Lake Victoria Region Superflock, we painted the haplotypes at all ancestry informative sites either red or blue for Congolese or Upper Nile ancestry, respectively (Supplementary Data 3). To do this, we extracted all 19 positions from the alignment that are polymorphic in LVRS cichlids and have different nucleotides in Congolese and Upper Nile taxa (Supplementary Data 3). Twenty-seven haplotypes (36%) show indications of recombination between the ancestral Congolese and Upper Nile lineage haplotypes. Recombination between the ancestral haplotype groups thus seems to have played an important role in generating *LWS* sequence diversity in LVRS cichlids. Our findings implicate the ancient hybridization event as the source of the unusually large genetic variation at the *LWS* gene.

The amino acid change at amino acid (AA) position 177 in the cichlid *LWS* gene, which corresponds to position 164 in the bovine *LWS* rhodopsin and to position 180 in the human red- and green-opsins, is important for spectral tuning (part of the so-called "Five-site rule"[5]). The AA coded by the variant found in the Upper Nile taxa shifts peak absorbance towards longer wavelength (red) by 7 nm compared to the AA coded by the Congolese variant[6]. Many species that have predominantly or exclusively class II haplotypes occupy deep or turbid water both of which correspond in Lake Victoria to a red-shifted light environment relative to clear shallow water. The hybridization-derived polymorphism in this gene, and specifically the arrival of class II alleles from the Upper Nile ancestor through hybridization, may have facilitated colonization of this extreme light environment early in the radiation: Rocky shore algae scrapers of genera *Neochromis* and *Mbipia* and mud bottom detritivores of genus *Enterochromis* occupy opposite ends in the spectrum of ambient lights and visual ecologies in Lake Victoria: clear and shallow versus murky and deep. All 438 previously published *LWS* sequences from algae scrapers are part of haplotype class I (likely of Congolese lineage ancestry) or recombinants between the haplotype classes (see below), whereas 11 of the 12 previously published *Enterochromis LWS* sequences are either part of haplotype class II (likely of Upper Nile lineage ancestry) or are recombinants between the two haplotype classes[7]. Recombination

between haplotype classes seems to have facilitated fine-scale adaptation, associated with speciation, in both genera: The mostly clear-water adapted *Neochromis* lineage has two haplotypes (H and L) which are important in adaptation to clearer versus more turbid shallow water associated with incipient speciation[6]. Position 177 differs between the H and L haplotypes leading to higher red-sensitivity in carriers of the L haplotype (Supplementary Data 3). Whereas the clear-water H haplotype belongs to class I, the L haplotype seems to be a recombinant between the two haplotype classes (Supplementary Data 3). Within *Neochromis greenwoodi,* the L haplotype is associated with populations living in shallow but turbid habitats, where dispersed particulate matter absorbs light of short wavelengths, leading to a red shifted photic environment[6]. Fixation or near-fixation of these *LWS* variants between very closely related populations has been shown to be driven by divergent natural selection between different light environments[6]. Similarly, divergence within *Enterochromis* into a deeper living species with red male nuptial coloration (*E. cinctus*) and a shallower living species with blue male coloration (*E. antleter*) was associated with a shift from the turbid and dark adapted class II haplotypes in the former to a recombinant haplotype in the latter in which the amino acid residue at position 179 changed (corresponding to bovine rhodopsin AA pos. 166, Supplementary Data 3, Supplementary Fig. 7).

It seems possible that the amino acid shift at position 177 in Congolese derived haplotypes, likely acquired through recombination with Upper Nile derived haplotypes, has allowed the clear water-adapted *Neochromis* lineage to expand into more turbid environments, and that the shift at position 179 in Upper Nile derived haplotypes, likely acquired through recombination with Congolese derived haplotypes, has allowed the deep-adapted *Enterochromis* lineage to invade more shallow environments, though the effect of the AA change at position 179 is not known.

The amino acid differences between ancestral haplotypes at positions 62 and 137 of the cichlid *LWS* gene also have known effects on light absorbance of the opsin protein, shifting peak absorbance by several nm (Supplementary Data 3). Finally, the amino acid change at position 191 of the cichlid *LWS* gene (AA pos. 178 in bovine rhodopsin) is expected to disrupt hydrogen bonds with nearby amino acids, which may lead to a shift in absorbance sensitivity[4]. Also the five additional non-synonymous substitutions that differ

between Congolese and Upper Nile taxa, and are recombined in various ways in the LVRS, are all located in transmembrane helices, suggesting that some of them may cause functional differences between opsin genes too.

Sorting of ancestral alleles

The ancestral hybridization event that we infer here may have facilitated subsequent adaptive radiation by more generally providing both ecologically relevant genetic variation, variation in traits important to mate choice, and reproductive incompatibilities that could be sorted differentially among newly arising species. We tested this hypothesis beyond the *LWS* gene by studying candidate loci for divergent selection or involved in reproductive isolation among species in the Lake Victoria radiation. We chose six species, sampled from exact sympatry, that represent the morphological and ecological diversity of the Lake Victoria radiation: *Pundamilia pundamilia* (blue male nuptial coloration, benthic insectivore-omnivore), *P. nyererei* (red planktivore-omnivore), *Harpagochromis vonlinnei* (blue piscivore), *Lipochromis melanopterus* (yellow paedophage), *Paralabidochromis chilotes* (blue benthic insectivore) and *Neochromis omnicaeruleus* (blue algae scraper).

Of the 12,890 SNPs in these species, 340 (3%) were outliers of exceptionally high global F_{ST} between the species (LV outiers, $p<0.05$, Fig 5a) and hence candidate loci for divergent selection or reproductive isolation. We identified both alleles present in the Congolese taxa at 1,053 of the 12,890 SNP sites, of which 83 (8%) were outliers in the Lake Victoria species (Fig. 5a, category 2). These alleles would also have been present as standing variation in the radiation ancestor without Upper Nile hybridization. However, 1,038 other sites had an alternative allele segregating in the Lake Victoria species that was only found in the Upper Nile taxa (Fig. 5a, category 3). Of these, 101 (10%) were Lake Victoria outlier loci. Even though some of these alleles may represent unsampled alleles also segregating in the Congolese lineage, these results indicate that the opportunity for divergence at a large proportion of the Lake Victoria outlier loci (101 of 340) was introduced through hybridization with the Upper Nile lineage.

Next, we tested whether sites fixed for alternative alleles in the Congolese and Upper Nile taxa were enriched for outlier loci among Lake Victoria species. This would be expected if sites fixed for alternative alleles in the parental lineages were more often involved in incompatibilities or were otherwise under stronger divergent selection than sites with alleles segregating within either of the parental lineages. We found 100 sites that were fixed for alternative alleles in Congolese and Upper Nile taxa but had both alleles present in Lake Victoria (Fig. 5a, category 4). Of these sites, 20% were significant high global F_{ST} outliers among the six Lake Victoria species. As these hybridization-derived polymorphisms may have had a higher starting allele frequency compared to ancestral polymorphisms segregating at lower frequencies within the parental lineages, they may have been easier targets for diversifying selection. Just prior to the onset of the radiation in Lake Victoria we would estimate the minor allele frequency for these SNPs as ~16%, corresponding to the Upper Nile ancestry proportion in Lake Victoria cichlids (Supplementary Fig. 6, Supplementary Table 4). To test if the higher starting allele frequency in the Lake Victoria radiation alone could explain the enrichment for Lake Victoria outliers of sites fixed for alternative alleles in the Congolese and Upper Nile relatives of the radiation, we created a dataset of sites with comparable expected ancestral allele frequencies in the Lake Victoria radiation that were not fixed in the parental lineages. We approximated the ancestral allele frequency for the Lake Victoria radiation at each SNP as the weighted ancestral allele frequency, *i.e.* by multiplying the allele frequencies in the Congolese and Upper Nile taxa by 0.84 and 0.16, respectively. We then extracted all SNPs with a weighted minor allele frequency of 14-18%. We obtained 179 sites including 18 Lake Victoria high global F_{ST} outliers (Fig. 5a, category 5). The dataset of sites fixed for alternative alleles in the parental lineages contains a significantly higher proportion of Lake Victoria outliers compared to this dataset (0.2 vs 0.1, two-sided Fisher's exact test: p-value = 0.017). The same holds true if the test was repeated assuming an Upper Nile ancestry proportion of 10% to 30% (LV outlier proportions in all datasets ranging from 8-12%, data not shown).

Sites fixed for alternative alleles in the Congolese and Upper Nile lineage may be enriched for LV outliers because of some features of these genomic sites increasing their probability to become fixed in general (e.g. due to variation in mutation or recombination rates or purifying selection). To test for that, we

checked if LV outliers fixed for alternative alleles in the parental lineages were also between other species more often fixed for alternative alleles than non-outliers. We used all species from outside the radiation for which we had at least three sequenced individuals (*Astatotilapia bloyeti, A. sparsidens, A. flavijosephi, A. calliptera, A. burtoni,* and *Astatoreochromis alluaudi*, Supplementary Table 5). For each pairwise comparison, we extracted sites with at least three individuals sequenced at a minimum depth of six reads each in both species (Supplementary Table 5). We then checked if these sites were differentially fixed between the two control group species, *i.e.* if all individuals of one species had one allele and all individuals of the other species had another allele. We tested if the proportion of differentially fixed sites was different in the LV outliers than in non-outliers using a Fisher's exact test for count data. The tests were non-significant in all 16 pairwise comparisons ($p>0.45$ in all comparisons, Supplementary Table 5). This indicates that a generally increased fixation probability cannot explain the enrichment of LV outliers at sites fixed for alternative alleles between the parental lineages.

Together, these results indicate that sites that were fixed for alternative alleles in the parental lineages of the radiation are functionally different from those ancestral polymorphisms that segregated within either of the parental lineages, suggesting that ancestrally fixed sites are more often linked to adaptive divergence, incompatibilities or behavioural reproductive isolation. Except for two sites that are within 10 bp from each other, all such sites that we found are on different reference genome scaffolds indicating that such sites are widely distributed across the genome. Sites fixed for alternative alleles in the Congolese and Upper Nile taxa which are high global F_{ST} outliers in Lake Victoria are differentially sorted in different species within Lake Victoria. Figure 4b shows that the high global F_{ST} at these sites is not driven by a single species that differs at all sites from the others or by species differences in overall Congolese and Upper Nile ancestry proportions, but rather that at each site each species (almost) fixed one or the other parental allele in a mosaic fashion.

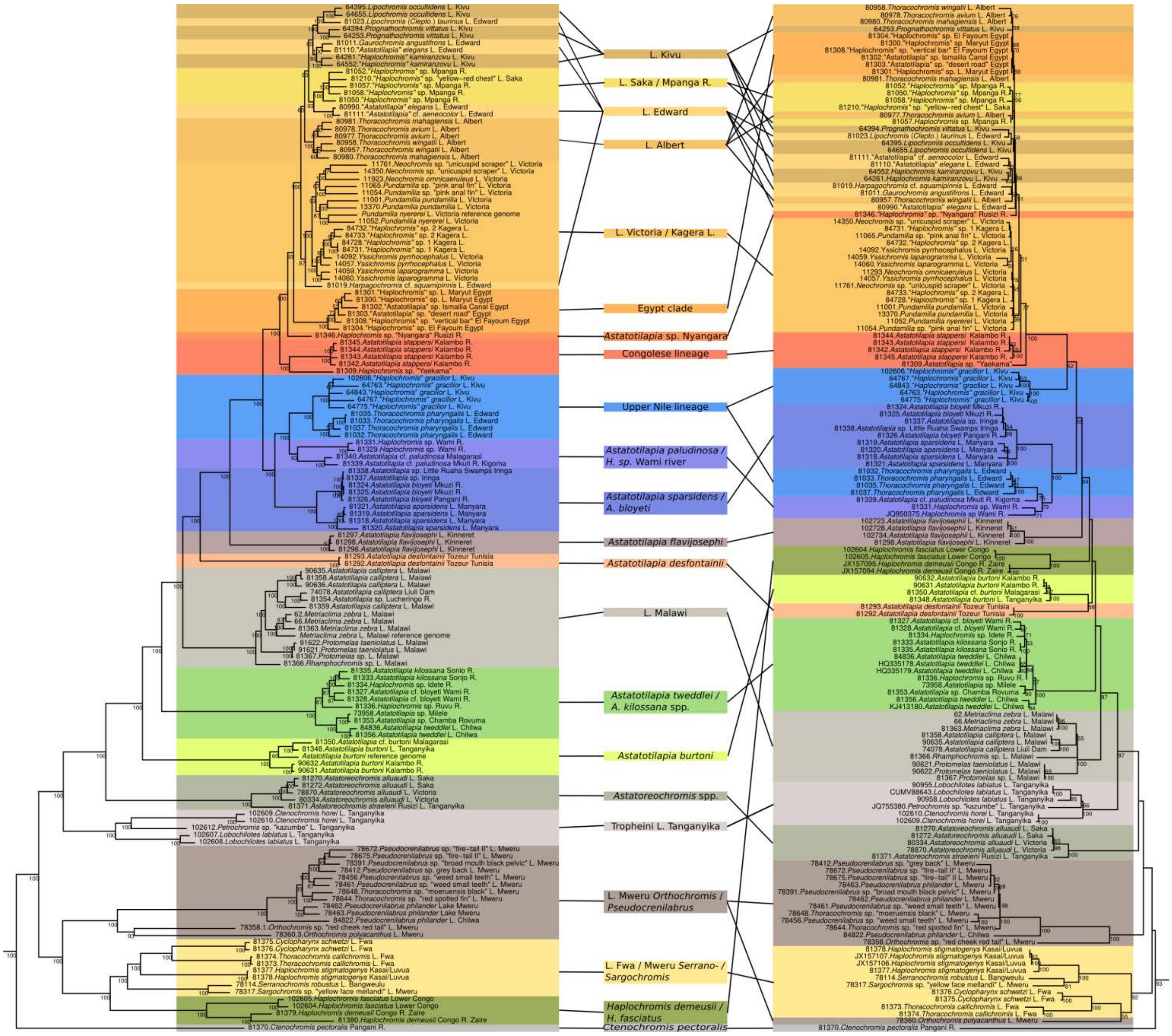

Supplementary Figure 1: Phylogenetic tree comparison. Maximum likelihood trees of concatenated RAD sequences (left) contrasted with the phylogeny reconstructed from mitochondrial sequences (right, Dloop and ND2). Bootstrap support values greater than 50 are shown below the respective nodes.

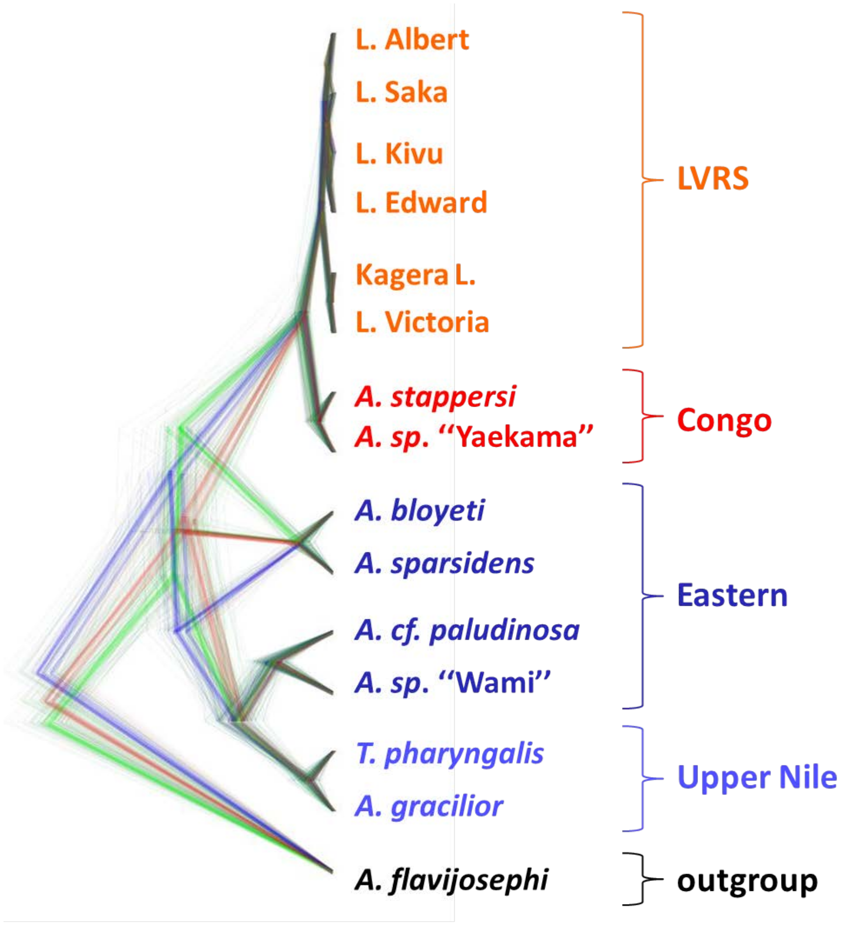

Supplementary Figure 2: SNAPP tree showing the three major consensus species trees with taxa used for the D statistics. SNAPP infers the species tree directly from SNPs, bypassing gene trees[8]. In the posterior distribution of species trees, 40.4% of the trees support the blue topology consistent with the RAxML tree, 31.6% the green, and 28% the red topology. Importantly, the Congolese taxa are always sister to the LVRS and the Upper Nile taxa are always nested within the Eastern clades.

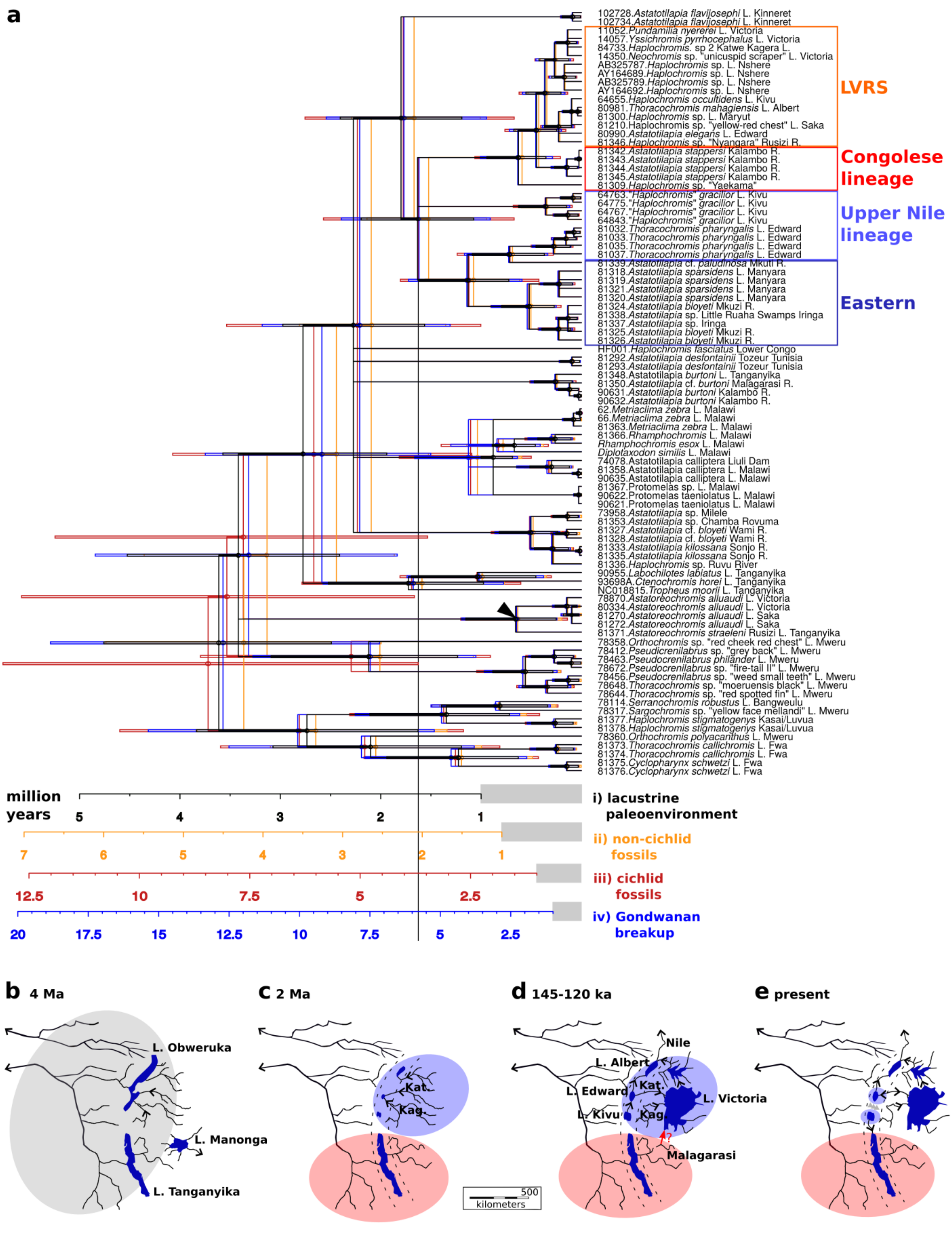

Supplementary Figure 3: Comparison of divergence times with paleogeographic events. (**a**) Maximum clade credibility trees reconstructed with BEAST[9] using four different sets of calibration (Supplementary Table 1) overlaid in different colours. The respective time scales in million years (Ma) are shown below the tree using the same colouring scheme. The vertical line indicates the divergence time between the Congolese and Upper Nile lineages. Nodes with a posterior probability of less than 70 are collapsed. Horizontal bars show the 95% highest posterior density (HPD) intervals around the mean ages. As phylogenetic tree inference methods assumes a time-constant clock, whereas in fact the clock rate decays exponentially up to one million years[10-12], ages of divergence events

younger than 1 Ma are strongly overestimated and cannot be read from our graph (indicated by grey rectangles over the time scales). Note, that in discordance with the nuclear phylogeny, "*Haplochromis" gracilior* of the Upper Nile lineage is highly divergent from the other Upper Nile lineage taxon, *Thoracochromis pharyngalis*, whereas *T. pharyngalis* is placed sister to *Astatotilapia* cf. *paludinosa* consistent with the nuclear phylogeny (see Supplementary Discussion).(**b-e**) Paleogeographic maps based on previously published data and reviews[1,2,13-20]. Ellipses show the potential distribution range of the Congolese lineage, the Upper Nile lineage, and their common ancestor in red, blue and light grey, respectively. (**b**) In the early Pliocene, 4 Ma, Paleolake Obweruka had formed at the location of modern lakes Albert and Edward[15]. Like Lake Tanganyika, which also existed at this time, this paleolake drained into the Congo. (**c**) In the early Pleistocene, by 2 Ma, rifting and flank uplift along the Western branch of the East African Rift System had truncated the paleoriver network, separating the Lake Victoria Region from the Congo drainage system[13,18] and the Rwenzori Massif uplift had split paleo-Lake Obweruka into two smaller lakes[15]. About 400 thousand years ago (ka, time poorly constrained) regional tilting and further uplifting west of the present Lake Victoria lead to reversal of the flow direction of the Katonga (Kat.) and the Kagera (Kag.) Rivers, causing the filling of the Victoria basin, forming proto-Lake Victoria[21] which flowed over into the Nile[14]. Until today, the Lake Victoria region contains the headwaters of the Nile. During the time period between 145-15 ka, East Africa experienced several cycles of extremely arid and more humid periods[16,19]. In at least one of the more humid periods (e.g. ~145-120 ka[16,19,20] (**d**)), the Lake Victoria Region (Nile drainage system) was likely reconnected with the Congo drainage system through capture of some of the northern tributaries of the Malagarasi, creating opportunity for the colonization of the Upper Nile catchment by Congolese taxa and associated hybridization between the Congolese and Upper Nile lineages of haplochromine cichlids. A connection between the Lake Victoria Region and the Malagarasi at about that time is also supported by the unique sharing between the Tanganyika and LVR drainage systems of the distantly related haplochromine genus *Astatoreochromis* and the sequence divergence between the Tanganyika endemic *A. straeleni* and the LVR endemic *A. alluaudi* (indicated with a black triangle). Additional evidence for connections is the presence of fish in the Malagarasi with Victoria or Nile origin (de Vos *et al.*, 2001) and vice versa (Seehausen *et al.*, 2002). (**e**) Since the eruption of the Virunga volcanoes (grey triangles), around 11-25 ka[1,2], the outflow of Lake Kivu to the North (Lake Edward) is blocked and the lake now drains southward through the Rusizi river into Lake Tanganyika, and thus into the Congo drainage system. Lake Victoria is connected to Lake Albert via the Victoria Nile, but the Murchison Falls prevent upstream fish passage. Lakes Albert and Edward are connected by the Semliki River, but rapids pose a barrier to upstream fish movement.

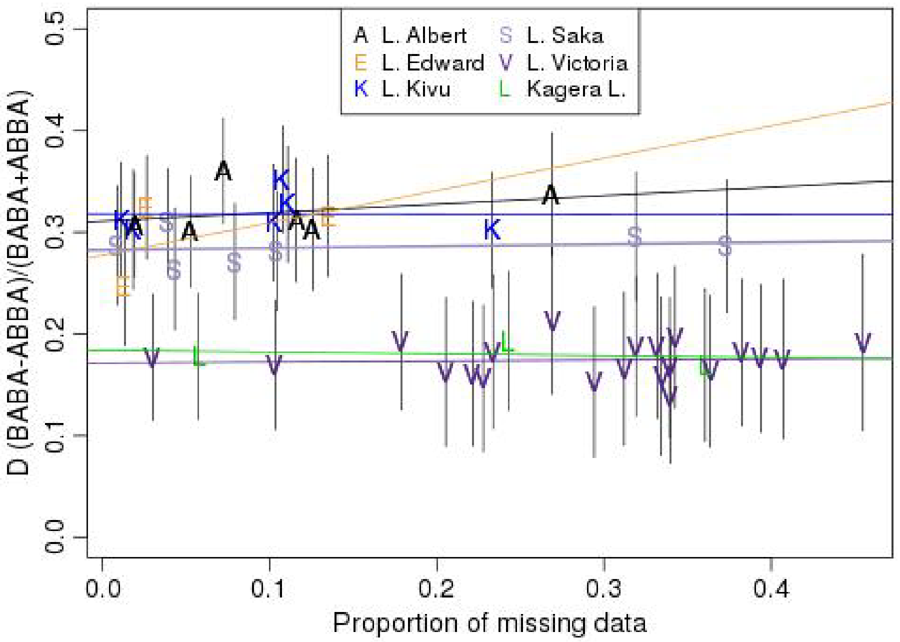

Supplementary Figure 4: Variation of D statistics among individuals and correlation with missing data proportion. D statistics are not biased by missing data. D statistics for the test shown in the manuscript Fig. 2 using each LVRS individual separately as P1 are plotted against the respective missing data proportion of the P1 individual (P2=*A. stappersi*, P3=*H. gracilior*, outgroup=*A. flavijosephi*). Vertical bars correspond to three standard errors from the mean, coloured lines show correlation of D statistics with missing data for each lake separately (all correlations are non-significant).

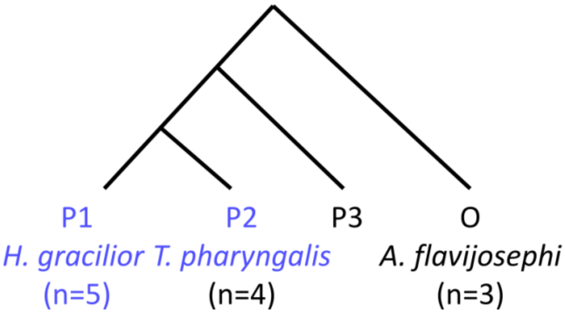

P3	Dstat	z
Lake Victoria (n=21)	0.00	-0.30
Lake Edward (n=3)	0.00	0.21
Lake Albert (n=6)	0.01	0.43
Lake Kivu (n=6)	0.02	1.50
Lake Saka (n=7)	0.00	0.16

Supplementary Figure 5: “*Haplochromis*” *gracilior* and *Thoracochromis pharyngalis* share equal proportions of alleles with each LVRS group. The genealogy tested is shown on the left and D and z values for each ABBA-BABA test using different LVRS groups as P3 are shown in the table. Positive D values with z > 3 would suggest that BABA is more frequent than ABBA and thus *H. gracilior* shares more alleles with P3 than *T. pharyngalis*. Negative D values with z < -3 would suggest excess allele sharing between P3 and *T. pharyngalis* relative to *H. gracilior* (ABBA more frequent than BABA). Here, all D values are around 0, with absolute z-scores below 3, which means that all lake radiations share the same proportion of alleles with *H. gracilior* and with *T. pharyngalis*. This implies that the signal of gene flow between LVRS and Upper Nile taxa (Fig. 2) is not explained by recent gene flow in current sympatry between LVRS members and *H. gracilior* in Lake Kivu or LVRS members and *T. pharyngalis* in Lake Edward. Number of individuals (n) used for each taxon are indicated in parentheses. All tests are based on allele frequencies and were performed with ADMIXTOOLS v. 1.1[22].

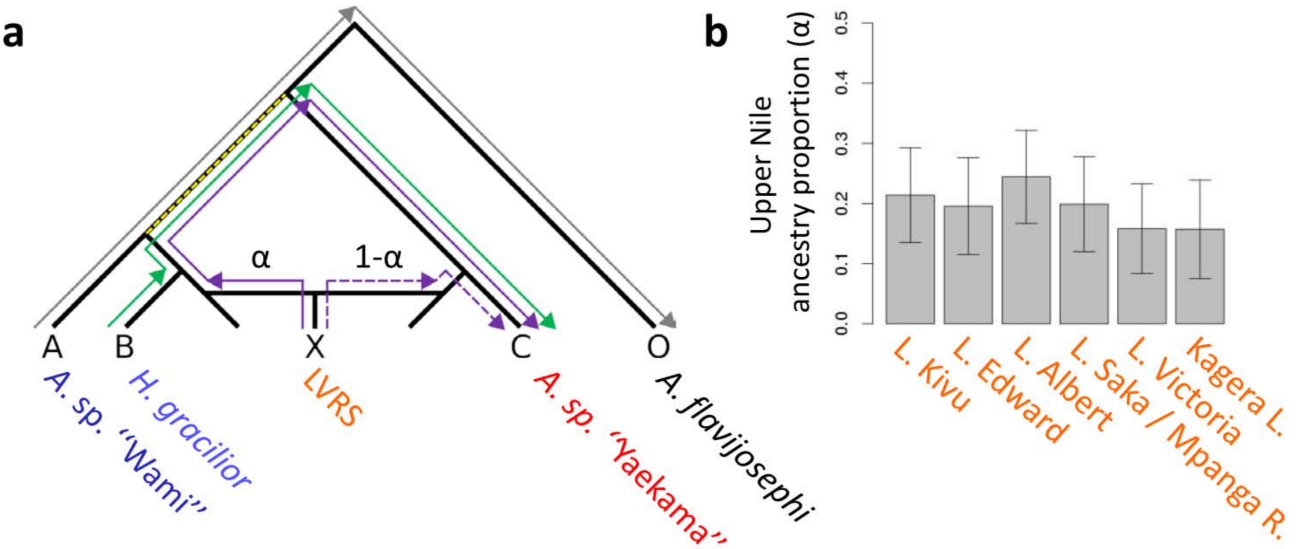

c

$$\alpha = \frac{\sum_{i=1}^{n}(p_i^{Wami} - p_i^{flavijosephi})(p_i^{LVRS} - p_i^{Yaekama})}{\sum_{i=1}^{n}(p_i^{Wami} - p_i^{flavijosephi})(p_i^{gracilior} - p_i^{Yaekama})}$$

Supplementary Figure 6: The Upper Nile ancestry proportion in the LVRS estimated with F4-ratio tests is about 20%. (**a**) Phylogeny used for the F4-ratio tests to calculate the Upper Nile ancestry proportion in the LVRS. The F4-ratio test makes the assumption that allele frequencies change only through drift[22]. Allele frequencies in two populations are thus more similar the less opportunity for drift was present along their branches to their common ancestor (shorter drift paths). Relevant drift paths are indicated with arrows matching the colour of the respective multiplicands in the F4-ratio test formula (c). The branch which is shared by all drift paths is highlighted with yellow dashes. (**b**) Estimated Upper Nile ancestry proportions (α) for each LVRS group. Error bars are three standard errors from α. Exact values and additional tests with *Thoracochromis pharyngalis* as population B and other A groups are given in Supplementary Table 4. (**c**) F4-ratio test formula to calculate the Upper Nile ancestry proportion from allele frequency (p) differences over n sites. The F4-ratio test is based on correlations between allele frequency differences between two pairs of populations. Allele frequency differences between two pairs of populations will be correlated if one of the populations shares a drift path (common ancestry) with a population of the other pair. As an example, if we take the genealogy of populations (((A, B), C), O) shown in panel a, the allele frequency differences between A and O would be correlated with the allele frequency differences between B and C because of the shared drift path between A and B (yellow-black dashed line). If e.g. at a given site the ancestral minor allele frequency at the root was low but increased through drift along the yellow-dashed branch, both A and B would have increased allele frequencies as they both share this yellow-dashed branch. Therefore, both the allele frequency differences between A and O and between B and C would increase. The magnitude of correlation between these two allele frequency differences is proportional to the shared drift path. If population B was of hybrid origin, it would only partially share the yellow-dashed drift path with A and the correlation between the allele frequency differences would weaken to the extent of allelic contribution of the other parental lineage. Here, the Wami River cichlids and "*Haplochromis*" *gracilior* share the branch dashed in yellow which leads to correlated allele frequency differences between the Wami River sp. and *Astatotilapia flavijosephi* (grey) and between *A.* sp. "Yaekama" and *H. gracilior* (green). This can be seen by the overlap of the grey and green drift paths along the yellow-dashed branch in (a). If an LVRS group was completely of Upper Nile origin (ancestor was a sister group of *H. gracilior*), all allele frequency differences between the LVRS group and *A*. sp. "Yaekama" (purple) would be due to drift along the drift paths indicated with solid purple arrows. The overlap of the drift paths between the grey and purple arrows would be equal to that of the grey and green arrows (at the yellow branch) leading to a ratio of f4 statistics of α = 1. If the LVRS group was completely derived from a Congolese lineage sister to *A*. sp. "Yaekama", the allele frequency differences between LVRS and *A*. sp. "Yaekama" would be uncorrelated with the allele frequency difference between the Wami River cichlids and *A. flavijosephi* (no shared path between dashed purple arrows and grey arrows). The numerator of the F4-ratio test would equal to 0 and thus the Upper Nile ancestry proportion (α) would be 0. Here, each LVRS group is admixed with partial Congolese and partial Upper Nile ancestry indicated by α which corresponds to the Upper Nile ancestry proportion in the LVRS group which is proportional to the overlap of the solid purple and grey.

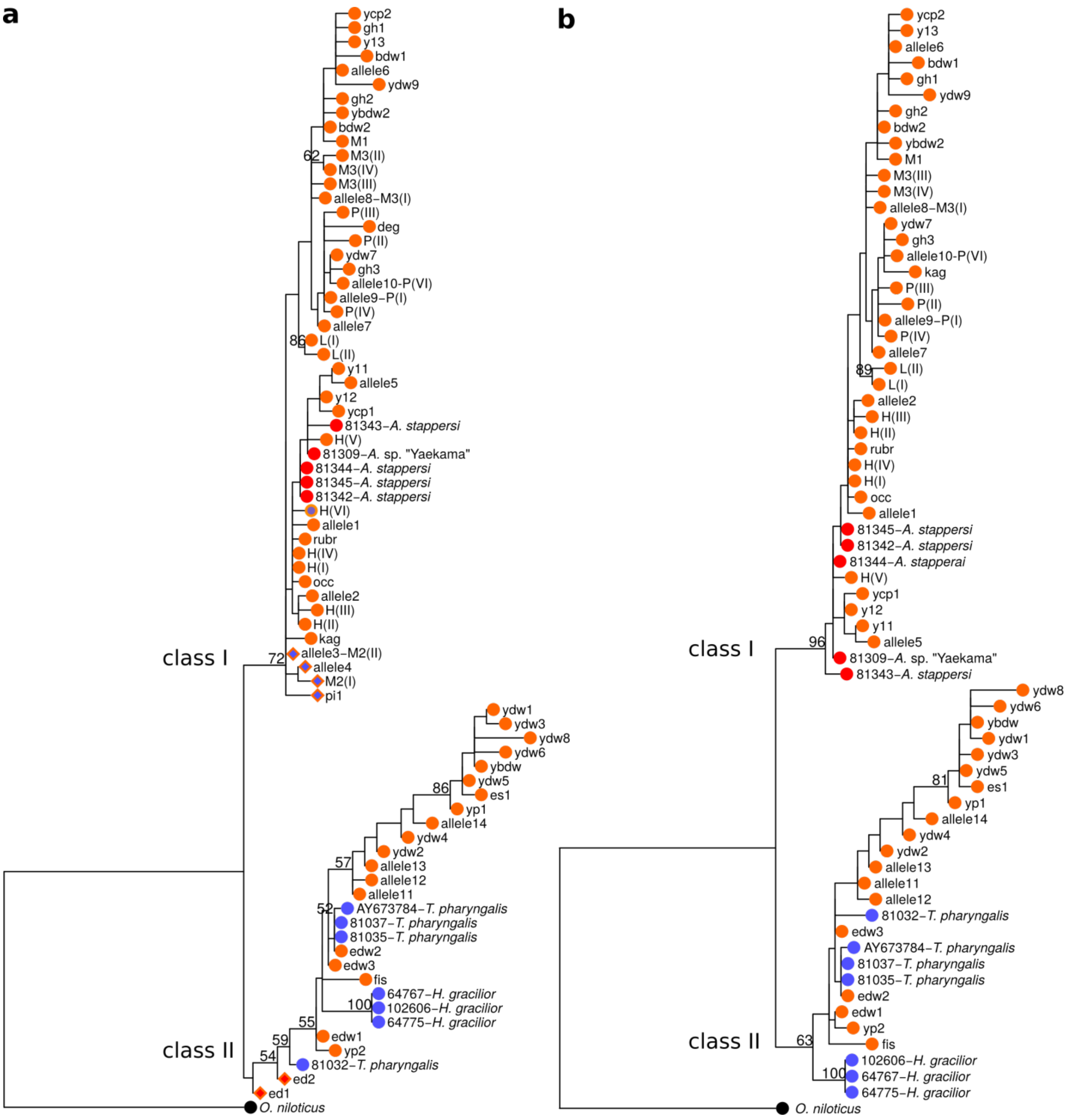

Supplementary Figure 7: Each of the two major *LWS* opsin haplotype classes is shared exclusively with either the Congolese or the Upper Nile lineage. (**a**) RAxML tree with all known LWS (long-wave sensitive opsin) haplotypes[4,6,7,23] and additional new sequences, found in the LVRS (orange) and those found in the Congolese (red) and Upper Nile lineage (blue). Recombinant sequences removed in (**b**) (and in Fig. 4) are indicated with bi-colored squares. For known occurrences in LVRS species and references, see Supplementary Data 3 and Supplementary Discussion.

a

Sample	n	P	Missing data prop.	mean depth	f_d	D (z)
Pundamilia pundamilia (Lake Victoria)	4	P2	3.0%	19.2	0.09	0.22 (40.3)
Pundamilia nyererei (Lake Victoria)	4	P2	3.0%	18.8	0.09	0.22 (41.8)
Paralabidochromis flavus (Lake Victoria)	1	P2	3.1%	15.7	0.08	0.23 (42.0)
Harpagochromis vittatus (Lake Kivu)	1	P2	3.0%	16.3	0.13	0.34 (70.4)
Paralabidochromis paucidens (Lake Kivu)	1	P2	2.7%	16.1	0.16	0.37 (74.9)
"Haplochromis" gracilior (Upper Nile)	1	P3	3.3%	15.6		
Thorachromis pharyngalis (Upper Nile)	1	P3	3.7%	14.8		
Astatotilapia stappersi (Congolese)	1	P1	10.3%	9.8		

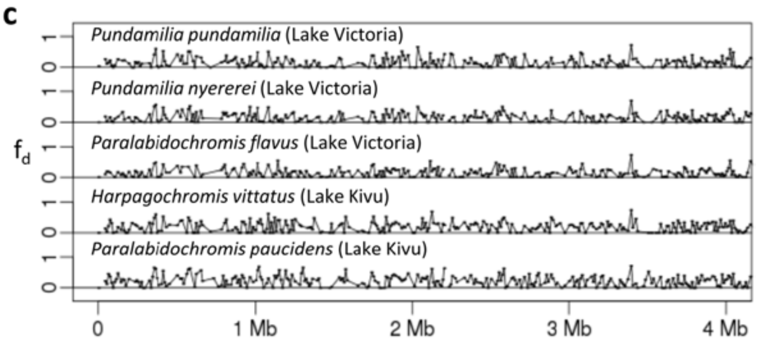

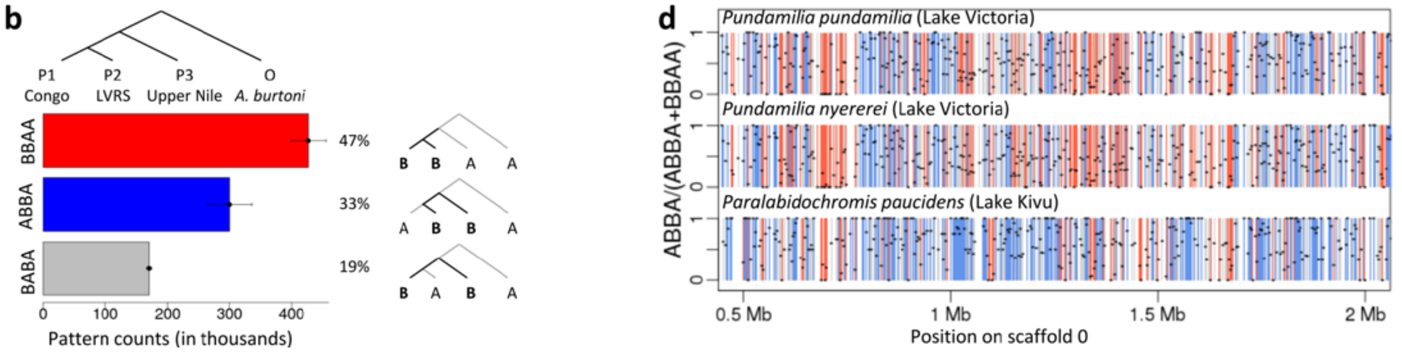

Supplementary Figure 8: Whole-genome sequencing data support an ancient hybridization event at the base of the Lake Victoria Region Superflock. (**a**) Number of individuals (n) per species, population label used for f_d statistics, mean missing data proportion, and mean depth of coverage for all samples. f_d and D values with associated z-scores based on 7,382,197 bi-allelic SNPs are given for all LVRS taxa. (**b**) Mean total counts of the phylogeny-concordant BBAA pattern and of the two possible discordant (ABBA and BABA) patterns across the five species. In a scenario of ILS, both discordant patterns are expected at similar frequencies. We find a highly significant excess of ABBA over BABA. Note, that as shown in the genealogy on top, LVRS taxa are used as P2 to calculate f_d (in contrast to D stats [see Fig. 2], where LVRS taxa were used as P1 and the Congolese lineage as P2). This difference is simply for consistency with the description of f_d in Martin *et al.*[24], and thus here ABBA represents sites with a derived allele shared between LVRS and Upper Nile, and BABA represents sites with a derived allele shared between Congolese and Upper Nile lineage taxa. Positive f_d values indicate gene flow between P3 (Upper Nile) and P2 (LVRS). (**c**) f_d values of 10 kb windows do not reveal large ancestry blocks, consistent with a hybridization event several thousand generations ago. Here, we show the first 4 Mb of the first scaffold (scaffold 0) which is representative of the general pattern found across scaffolds. (**d**) To visualize putative ancestry tracts at a higher resolution, we counted sites where LVRS taxa share a derived allele with either the Upper Nile lineage (ABBA) or the Congolese lineage (BBAA). The proportion of phylogeny-discordant ABBA sites out of the sum of ABBA and BBAA patterns is shown for 3 kb non-overlapping windows using the four *Pundamilia pundamilia* individuals (top), the four *P. nyererei* (middle), or *Haplochromis vittatus* (bottom) as P2. Genomic regions with a minimum ABBA proportion of 0.7 are coloured in blue (putatively Upper Nile derived), genomic regions with maximum ABBA proportion of 0.3 are coloured in red (putatively Congolese derived). Regions with intermediate frequencies of ABBA and BBAA (likely containing a mix of Congolese and Upper Nile derived sites) are coloured in grey.

Supplementary Table 1: Mitochondrial chronogram calibration sets and age estimates. Calibration sets used for inference of the dated mitochondrial trees and estimates obtained for the age of the divergence event between the Upper Nile lineage and the Congolese lineage (Congo). All dates are given in million years. Dated trees obtained with the four different calibration sets are shown in Supplementary Fig. 3.

	**i) lacustrine paleoenviron-mental calibration**	**ii) non-cichlid fossils calibration**	**iii) cichlid fossil calibration**	**iv) Gondwanan fragmentation calibration**
References used for calibration	Sturmbauer *et al.*, 2001[25]<br>Koblmüller *et al.*, 2008[26]	Friedman *et al.*, 2013[27]<br>Genner *et al.*, 2015[28]	Genner *et al.*, 2007[12]	Genner *et al.*, 2007[12]<br>Genner *et al.*, 2015[28]
„modern haplo-chromines“ prior	Normal prior on crown age, mean 3.4, SD 0.5, 95% CI 2.58-4.22	Normal prior on crown age (exclu-ding Tropheini), mean 2.8, SD 0.5, 95% CI 1.98-3.62	Normal prior on stem age, mean 8.9, SD 2.3, 95% CI 5.12-12.7	Normal prior on crown age (exclu-ding Tropheini), mean 8.5, SD 2, 95% CI 5.2-11.8
Lake Malawi radiation crown age prior	Uniform prior, 0.57-1 (refilling of Lake Malawi)	Normal prior, mean 1.2, SD 0.5, 95% CI 0.378-2.02	Normal prior, mean 2.2, SD 1.2, 95% CI 0.22-4.17	Normal prior, mean 4.06, SD 1, 95% CI 1.9-6.2
Upper Nile vs Congo/LVRS split	1.63 [1.07,2.20]	1.92 [1.19,2.69]	3.69 [1.53,5.84]	5.79 [3.26,8.56]

Supplementary Table 2: D statistics with different P2 and P3 taxa, different outgroups and reference genomes. D statistics and z-values for different combinations of P1 (LVRS groups shown in different columns), P2 (Congolese taxa), P3 (Eastern clade or Upper Nile taxa) and outgroups and using either *Pundamilia nyererei* (nyer) or *Astatotilapia burtoni* (burt) as reference genome for read mapping. For each analysis, individuals with less than 50% of the sites covered with at least 10 reads were excluded. Numbers of individuals retained are given in brackets. All tests are based on allele frequencies and performed with ADMIXTOOLS.

**D statistics with different P3 groups (165,920 SNPs) used for Fig. 2**				**D statistics (z-values) for different LVRS groups as P1**					
**ref**	**P2**	**P3**	**outgroup**	**L. Albert (6)**	**L. Edward (3)**	**L. Kivu (6)**	**L. Saka (7)**	**L. Victoria (21)**	**Kagera L. (3)**
nyer	*A. stappersi* (4)	*H. gracilior* (5)	*A. flavijosephi* (3)	0.32 (19.63)	0.29 (17.39)	0.32 (19.35)	0.29 (17.14)	0.18 (9.43)	0.17 (8.54)
nyer	*A. stappersi* (4)	*T. pharyngalis* (4)	*A. flavijosephi* (3)	0.31 (18.55)	0.29 (16.45)	0.30 (17.42)	0.29 (16.29)	0.18 (9.24)	0.17 (8.06)
nyer	*A. stappersi* (4)	*A. paludinosa* (2)	*A. flavijosephi* (3)	0.00 (0.22)	-0.02 (-1.18)	-0.02 (-1.12)	-0.02 (-0.91)	-0.03 (-1.33)	-0.03 (-1.52)
nyer	*A. stappersi* (4)	*A. bloyeti* (5)	*A. flavijosephi* (3)	-0.05 (-2.57)	-0.06 (-2.71)	-0.06 (-2.91)	-0.06 (-2.99)	-0.06 (-2.84)	-0.05 (-2.26)
nyer	*A. stappersi* (4)	*A. sparsidens* (3)	*A. flavijosephi* (3)	-0.04 (-2.18)	-0.05 (-2.27)	-0.05 (-2.53)	-0.06 (-2.83)	-0.05 (-2.62)	-0.05 (-2.54)
nyer	*A. stappersi* (4)	*H.* sp. ''Wami'' (2)	*A. flavijosephi* (3)	0.07 (3.18)	0.04 (1.78)	0.04 (2.08)	0.03 (1.35)	0.00 (-0.01)	0.00 (-0.01)
**Different Congolese taxon (*A.* sp. ''Yaekama '') as P2 (181,367 SNPs)**				**D statistics (z-values) for different LVRS groups as P1**					
**ref**	**P2**	**P3**	**outgroup**	**L. Albert (6)**	**L. Edward (3)**	**L. Kivu (6)**	**L. Saka (7)**	**L. Victoria (21)**	**Kagera L. (3)**
nyer	*A.* sp. 'Yaekama' (1)	*H. gracilior* (5)	*A. flavijosephi* (3)	0.41 (25.52)	0.39 (22.99)	0.41 (24.90)	0.39 (23.51)	0.29 (15.57)	0.28 (13.91)
nyer	*A.* sp. 'Yaekama' (1)	*T. pharyngalis* (4)	*A. flavijosephi* (3)	0.41 (25.04)	0.38 (22.47)	0.40 (23.91)	0.39 (22.63)	0.30 (16.11)	0.29 (13.78)
nyer	*A.* sp. 'Yaekama' (1)	*A. paludinosa* (2)	*A. flavijosephi* (3)	0.13 (6.66)	0.10 (5.02)	0.10 (5.17)	0.11 (5.67)	0.10 (5.24)	0.10 (4.59)
nyer	*A.* sp. 'Yaekama' (1)	*A. bloyeti* (5)	*A. flavijosephi* (3)	0.07 (2.94)	0.06 (2.58)	0.05 (2.28)	0.06 (2.59)	0.06 (2.81)	0.08 (2.97)
nyer	*A.* sp. 'Yaekama' (1)	*A. sparsidens* (3)	*A. flavijosephi* (3)	0.08 (3.27)	0.06 (2.69)	0.06 (2.44)	0.06 (2.71)	0.07 (2.88)	0.07 (2.57)
nyer	*A.* sp. 'Yaekama' (1)	*H.* sp. ''Wami'' (2)	*A. flavijosephi* (3)	0.19 (8.54)	0.16 (7.04)	0.17 (7.65)	0.16 (6.91)	0.14 (6.11)	0.13 (5.48)
**Different outgroups (206,810 SNPs)**				**D statistics (z-values) for different LVRS groups as P1**					
**ref**	**P2**	**P3**	**outgroup**	**L. Albert (6)**	**L. Edward (3)**	**L. Kivu (6)**	**L. Saka (7)**	**L. Victoria (11)**	**Kagera L. (3)**
nyer	*A. stappersi* (4)	*H. gracilior* (5)	*A. burtoni* (4)	0.38 (28.17)	0.37 (27.32)	0.38 (28.57)	0.36 (26.07)	0.27 (17.09)	0.26 (15.79)
nyer	*A. stappersi* (4)	*H. gracilior* (5)	*A. desfontainii* (2)	0.36 (26.42)	0.35 (24.59)	0.35 (26.00)	0.34 (24.80)	0.24 (15.51)	0.23 (13.70)
nyer	*A. stappersi* (4)	*T. pharyngalis* (4)	*A. burtoni* (4)	0.37 (25.23)	0.37 (24.77)	0.36 (24.81)	0.35 (23.85)	0.26 (15.40)	0.25 (14.08)
nyer	*A. stappersi* (4)	*T. pharyngalis* (4)	*A. desfontainii* (2)	0.35 (24.26)	0.34 (21.62)	0.33 (22.95)	0.33 (22.29)	0.23 (13.86)	0.22 (12.15)
nyer	*A. stappersi* (4)	*A. paludinosa* (1)	*A. burtoni* (4)	0.11 (5.85)	0.10 (5.20)	0.08 (4.39)	0.09 (4.51)	0.09 (4.42)	0.07 (3.39)
nyer	*A. stappersi* (4)	*A. paludinosa* (1)	*A. desfontainii* (2)	0.07 (3.65)	0.05 (2.58)	0.03 (1.60)	0.05 (2.42)	0.04 (2.05)	0.03 (1.24)
nyer	*A. stappersi* (4)	*A. bloyeti* (5)	*A. burtoni* (4)	0.06 (3.23)	0.06 (3.03)	0.04 (2.06)	0.05 (2.70)	0.05 (2.66)	0.05 (2.55)
nyer	*A. stappersi* (4)	*A. bloyeti* (5)	*A. desfontainii* (2)	0.02 (0.88)	0.01 (0.36)	0.00 (-0.25)	0.01 (0.50)	0.00 (0.25)	0.00 (0.23)
nyer	*A. stappersi* (4)	*A. sparsidens* (3)	*A. burtoni* (4)	0.05 (3.00)	0.06 (3.15)	0.03 (1.83)	0.05 (2.50)	0.05 (2.50)	0.05 (2.23)
nyer	*A. stappersi* (4)	*A. sparsidens* (3)	*A. desfontainii* (2)	0.01 (0.35)	0.00 (0.17)	-0.02 (-0.87)	0.00 (-0.01)	-0.01 (-0.51)	-0.01 (-0.55)
nyer	*A. stappersi* (4)	*H.* sp. ''Wami'' (2)	*A. burtoni* (4)	0.16 (8.85)	0.15 (7.65)	0.15 (7.62)	0.13 (6.80)	0.10 (5.10)	0.09 (4.39)
nyer	*A. stappersi* (4)	*H.* sp. ''Wami'' (2)	*A. desfontainii* (2)	0.13 (7.17)	0.11 (5.63)	0.11 (5.80)	0.10 (5.26)	0.06 (3.24)	0.05 (2.48)
**Different reference genome (117,712 SNPs)**				**D statistics (z-value) for different LVRS groups as P1**					
**ref**	**P2**	**P3**	**outgroup**	**L. Albert (3)**	**L. Edward (0)**	**L. Kivu (2)**	**L. Saka (3)**	**L. Victoria (12)**	**Kagera L. (2)**
burt	*A. stappersi* (4)	*H. gracilior* (3)	*A. flavijosephi* (1)	0.33 (19.63)	na	0.29 (14.92)	0.28 (14.86)	0.18 (8.94)	0.19 (8.79)
burt	*A. stappersi* (4)	*T. pharyngalis* (2)	*A. flavijosephi* (1)	0.33 (18.23)	na	0.28 (13.39)	0.29 (14.50)	0.17 (8.13)	0.18 (8.08)
burt	*A. stappersi* (4)	*A. bloyeti* (2)	*A. flavijosephi* (1)	0.02 (1.11)	na	-0.03 (-1.29)	-0.02 (-0.76)	-0.01 (-0.49)	0.00 (0.16)
burt	*A. stappersi* (4)	*A. sparsidens* (2)	*A. flavijosephi* (1)	0.02 (1.12)	na	-0.02 (-0.83)	0.00 (-0.19)	-0.01 (-0.28)	0.00 (0.14)

**Egypt clade and A. sp. "Nyangara"**				**D statistics (z-value) for different groups as P1**	
**ref**	**P2**	**P3**	**outgroup**	**Egypt clade (6)**	***A.* sp. "Nyangara" (1)**
nyer	*A. stappersi* (4)	*H. gracilior* (3)	*A. flavijosephi* (1)	0.4 (24.6)	0.09 (3.9)
nyer	*A. stappersi* (4)	*T. pharyngalis* (2)	*A. flavijosephi* (1)	0.4 (24.9)	0.08 (3.5)

Supplementary Table 3: Five-population test results. Schematic description of the four five-population D statistics with discordant allele sharing patterns and formulae for the different D statistics (D12, D1 and D2 are from Eaton and Ree[29]). The topology on top left shows the naming scheme of the tested populations. Most bi-allelic sites will show allele sharing patterns in concordance with this tree (e.g. P1 + P2 share a derived allele (B), whereas the other taxa share the ancestral allele (A) with the outgroup (BBAAA)). Due to incomplete lineage sorting or gene flow, some sites will show discordant allele sharing patterns such as those illustrated with the topologies on the right. "A" denotes the allele found in the outgroup (putative ancestral allele, black branches in the topologies) and "B" denotes the alternative allele (grey branches). For the five-population test, sites showing the discordant allele sharing patterns are counted to calculate the four D statistics (D12, D1, D2 and D21) shown in the columns on top right. In the case of no gene flow, the upper and the lower allele sharing patterns arise only due to incomplete lineage sorting and are expected to be equally frequent leading to a D value of 0. However, if the upper allele sharing pattern was more frequent then the bottom one, the respective D statistic would be positive. a-g, Predictions of D statistic values based on different gene flow scenarios with (a) no gene flow or (b-e) unidirectional gene flow as indicated by arrows and (f,g) ancestral gene flow (direction cannot be distinguished). Results below: observed mean D values, z scores in standard deviation units and mean pattern counts based on comparisons with the three individuals with least missing data for each of the Lake Victoria Region radiations (Albert, Kivu, Edward and Victoria), and the individual with the least missing data for each of the other groups: Congo: A. sp. "Yaekama" individual, Upper Nile: H. gracilior individual, Eastern: *A. bloyeti* individual and *A. flavijosephi* individual as outgroup. Significant values (|z|>3) are highlighted in bold. The results 1.1-1.4 indicate unidirectional gene flow from the Upper Nile clade into the LVRS groups (D12<0, D1<0, D2>=0, D21=0, matching predictions of scenario d). 2.1-2.4 suggest gene flow of a taxon equally distant to *H. gracilior* and *T. pharyngalis* (i.e. their ancestor or a close relative of both) with each LVRS group (D12=0, D1=0, D2=0, D21>0, matching predictions of scenario f). Alternatively, if e.g. *T. pharyngalis* had introgressed into the LVRS after it split from *H. gracilior*, and the elevated Patterson's D statistic for *H. gracilior* was only a side-effect due to high ancestral allele sharing between *H. gracilior* and *T. pharyngalis*, D1 would be negative and D2 potentially positive (scenario b). In contrast, if e.g. recent gene flow in current sympatry from the Lake Edward radiation had occurred into *T. pharyngalis*, D12 would be negative and D21 would be zero in line 2.2 (scenario d).

	$D12 = \frac{ABBBA-BABBA}{ABBBA+BABBA}$	$D1 = \frac{ABBAA-BABAA}{ABBAA+BABAA}$	$D2 = \frac{ABABA-BAABA}{ABABA+BAABA}$	$D21 = \frac{BBBAA-BBABA}{BBBAA+BBABA}$	
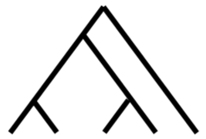	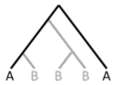	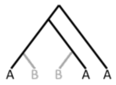	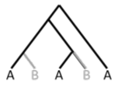	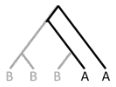	if more frequent D > 0
	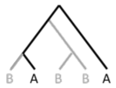	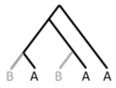	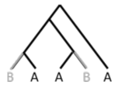	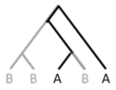	if more frequent D < 0

Predictions for different gene flow scenarios

		D12	D1	D2	D21
a	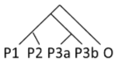	D12 = 0	D1 = 0	D2 = 0	D21 = 0
b	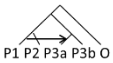	D12 = 0	D1 < 0	D2 >= 0	D21 > 0
c	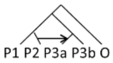	D12 = 0	D1 > 0	D2 <= 0	D21 > 0
d	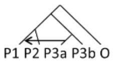	D12 < 0	D1 < 0	D2 >= 0	D21 = 0
e	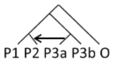	D12 > 0	D1 > 0	D2 <= 0	D21 = 0
f	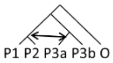	D12 = 0	D1 = 0	D2 = 0	D21 > 0
g	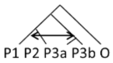	D12 < 0	D1 < 0	D2 < 0	D21 = 0

Results

Genealogy (((P1,P2),(P3a,P3b)),O)	D12 (z-score)	D1 (z-score)	D2 (z-score)	D21 (z-score)	counts
1.1 (((Albert, Congo),(Upper Nile, Eastern), *A. flavijosephi*)	**-0.28 (-8.3)**	**-0.51 (-17.4)**	**0.17 (4.6)**	0.07 (2.1)	417
1.2 (((Edward, Congo),(Upper Nile, Eastern), *A. flavijosephi*)	**-0.31 (-8.7)**	**-0.52 (-19.4)**	**0.24 (6.4)**	0.08 (2.6)	416
1.3 (((Kivu, Congo),(Upper Nile, Eastern), *A. flavijosephi*)	**-0.26 (-7.5)**	**-0.5 (-19.2)**	**0.2 (5.4)**	0.08 (2.6)	425
1.4 (((Victoria, Congo),(Upper Nile, Eastern), *A. flavijosephi*)	**-0.25 (-6.4)**	**-0.4 (-11.1)**	0.08 (1.9)	0.01 (0.3)	354
2.1 (((*T.pharyngalis, H.gracilior*),(Albert, Congo)), *A. flavijosephi*)	-0.03 (-0.74)	-0.02 (-0.5)	-0.05 (-1.03)	**0.38 (15.27)**	423
2.2 (((*T.pharyngalis, H.gracilior*),(Edward, Congo)), *A. flavijosephi*)	-0.04 (-1.03)	-0.06 (-1.58)	-0.03 (-0.6)	**0.33 (13.25)**	426
2.3 (((*T.pharyngalis, H.gracilior*),(Kivu, Congo)), *A. flavijosephi*)	-0.00 (-0.1)	0.01 (0.25)	-0.07 (-1.43)	**0.34 (12.36)**	415
2.4 (((*T.pharyngalis, H.gracilior*),(Victoria, Congo)), *A. flavijosephi*)	-0.02 (-0.69)	-0.08 (-1.87)	-0.04 (-0.83)	**0.17 (5.71)**	346

Supplementary Table 4: Ancestry proportions calculated with different Eastern clades as sister species to the Upper Nile representatives. The letters A, B, X, C, and O correspond to the populations used for the F4-ratio test[22] with the genealogy (((A,B),C),O) with a potentially admixed lineage X with ancestry proportions from B and C (visualized in Supplementary Fig. 6a). The ancestry proportion of B in X is calculated as the alpha value using the formula in Supplementary Fig. 6c.

**Nilotic ancestry proportion**					**B = *H. gracilior***			**B = *T. pharyngalis***		
**A**	**B**	**X**	**C**	**O**	**alpha**	**stderr**	**z**	**alpha**	**stderr**	**z**
A. bloyeti	*H. gracilior / T. pharyngalis*	L. Kivu	*H.* sp. “Yaekama”	*A. flavijosephi*	0.23	0.080	2.8	0.21	0.076	2.8
A. bloyeti	*H. gracilior / T. pharyngalis*	L. Edward	*H.* sp. “Yaekama”	*A. flavijosephi*	0.25	0.080	3.1	0.23	0.077	3.0
A. bloyeti	*H. gracilior / T. pharyngalis*	L. Albert	*H.* sp. “Yaekama”	*A. flavijosephi*	0.29	0.076	3.9	0.27	0.073	3.8
A. bloyeti	*H. gracilior / T. pharyngalis*	L. Saka	*H.* sp. “Yaekama”	*A. flavijosephi*	0.25	0.078	3.1	0.23	0.075	3.1
A. bloyeti	*H. gracilior / T. pharyngalis*	L. Victoria	*H.* sp. “Yaekama”	*A. flavijosephi*	0.25	0.072	3.4	0.23	0.069	3.4
A. bloyeti	*H. gracilior / T. pharyngalis*	Kagera L.	*H.* sp. “Yaekama”	*A. flavijosephi*	0.29	0.083	3.5	0.28	0.079	3.5
A. cf. *paludinosa*	*H. gracilior / T. pharyngalis*	L. Kivu	*H.* sp. “Yaekama”	*A. flavijosephi*	0.18	0.031	5.8	0.16	0.028	5.7
A. cf. *paludinosa*	*H. gracilior / T. pharyngalis*	L. Edward	*H.* sp. “Yaekama”	*A. flavijosephi*	0.17	0.032	5.3	0.16	0.030	5.3
A. cf. *paludinosa*	*H. gracilior / T. pharyngalis*	L. Albert	*H.* sp. “Yaekama”	*A. flavijosephi*	0.23	0.031	7.2	0.21	0.029	7.2
A. cf. *paludinosa*	*H. gracilior / T. pharyngalis*	L. Saka	*H.* sp. “Yaekama”	*A. flavijosephi*	0.19	0.031	6.1	0.18	0.029	6.1
A. cf. *paludinosa*	*H. gracilior / T. pharyngalis*	L. Victoria	*H.* sp. “Yaekama”	*A. flavijosephi*	0.17	0.030	5.5	0.15	0.028	5.6
A. cf. *paludinosa*	*H. gracilior / T. pharyngalis*	Kagera L.	*H.* sp. “Yaekama”	*A. flavijosephi*	0.16	0.033	4.9	0.15	0.030	4.9
A. sparsidens	*H. gracilior / T. pharyngalis*	L. Kivu	*H.* sp. “Yaekama”	*A. flavijosephi*	0.24	0.078	3.0	0.22	0.074	3.0
A. sparsidens	*H. gracilior / T. pharyngalis*	L. Edward	*H.* sp. “Yaekama”	*A. flavijosephi*	0.25	0.077	3.3	0.24	0.073	3.2
A. sparsidens	*H. gracilior / T. pharyngalis*	L. Albert	*H.* sp. “Yaekama”	*A. flavijosephi*	0.31	0.074	4.2	0.29	0.069	4.2
A. sparsidens	*H. gracilior / T. pharyngalis*	L. Saka	*H.* sp. “Yaekama”	*A. flavijosephi*	0.25	0.075	3.3	0.24	0.071	3.3
A. sparsidens	*H. gracilior / T. pharyngalis*	L. Victoria	*H.* sp. “Yaekama”	*A. flavijosephi*	0.25	0.072	3.5	0.23	0.067	3.5
A. sparsidens	*H. gracilior / T. pharyngalis*	Kagera L.	*H.* sp. “Yaekama”	*A. flavijosephi*	0.25	0.085	3.0	0.24	0.079	3.0
H. sp. “Wami”	*H. gracilior / T. pharyngalis*	L. Kivu	*H.* sp. “Yaekama”	*A. flavijosephi*	0.21	0.026	8.2	0.19	0.024	8.1
H. sp. “Wami”	*H. gracilior / T. pharyngalis*	L. Edward	*H.* sp. “Yaekama”	*A. flavijosephi*	0.20	0.027	7.3	0.18	0.024	7.3
H. sp. “Wami”	*H. gracilior / T. pharyngalis*	L. Albert	*H.* sp. “Yaekama”	*A. flavijosephi*	0.24	0.026	9.5	0.22	0.023	9.4
H. sp. “Wami”	*H. gracilior / T. pharyngalis*	L. Saka	*H.* sp. “Yaekama”	*A. flavijosephi*	0.20	0.026	7.5	0.18	0.024	7.6
H. sp. “Wami”	*H. gracilior / T. pharyngalis*	L. Victoria	*H.* sp. “Yaekama”	*A. flavijosephi*	0.16	0.025	6.4	0.14	0.022	6.4
H. sp. “Wami”	*H. gracilior / T. pharyngalis*	Kagera L.	*H.* sp. “Yaekama”	*A. flavijosephi*	0.16	0.027	5.8	0.14	0.025	5.8
H. sp. “Wami”	*H. gracilior / T. pharyngalis*	Egypt clade	*H.* sp. “Yaekama”	*A. flavijosephi*	0.41	0.03	11.7	0.38	0.03	11.7
H. sp. “Wami”	*H. gracilior / T. pharyngalis*	“Nyangara”	*H.* sp. “Yaekama”	*A. flavijosephi*	0.06	0.03	1.9	0.06	0.03	1.9

Supplementary Table 5: LV outliers that are divergently fixed between the Congolese and Upper Nile lineage do not have inherently increased fixation probabilities. The proportion of sites fixed divergently between species in a control group (species from outside the LVRS) is not higher in sites that are outliers of high global F_{ST} among the six Lake Victoria species (LV outliers) than in non-outlier sites. Only the 100 sites fixed for alternative alleles in the Congolese and Upper Nile taxa are used for the tests. The first and second columns indicate the *Astatoreochromis* (Ar) and *Astatotilapia* (At) species used for each test. The third and fourth columns show the number of total sites (max 100) and LV outliers amongst them (max 20), respectively, that are sequenced in at least 3 individuals in both species. The fifth and sixth columns show the number of total sites and of LV outliers amongst them, respectively, that are also fixed for alternative alleles in the two species. The seventh and eighth columns show the proportion of sites fixed differentially in the two control group species among the sequenced LV outliers and among the 80 sites that are not outliers in the Lake Victoria species. The last column shows the p-values for the two-sided Fisher's exact test for count data.

		**Number of total sites sequenced (n>3)**	**Number of LV outliers sequenced (n>3)**	**Number of sites diff. fixed**	**Number of LV outliers diff. fixed**	**Proportion of LV outliers diff. fixed**	**Proportion of non-outliers diff. fixed**	**p-value Fisher's exact test**
Ast. bloyeti	*Ast. flavijosephi*	53	8	15	2	0.25	0.29	1.00
Ast. bloyeti	*Asr. alluaudi*	71	14	19	2	0.14	0.30	0.51
Ast. bloyeti	*Ast. burtoni*	79	13	20	3	0.23	0.26	1.00
Ast. bloyeti	*Ast. calliptera*	82	15	19	3	0.20	0.24	1.00
Ast. bloyeti	*Ast. spasidens*	74	13	8	1	0.08	0.11	1.00
Ast. flavijosephi	*Asr. alluaudi*	45	8	6	1	0.13	0.14	1.00
Ast. flavijosephi	*Ast. burtoni*	50	6	8	0	0.00	0.18	0.58
Ast. flavijosephi	*Ast. calliptera*	53	8	9	0	0.00	0.20	0.59
Ast. flavijosephi	*Ast. sparsidens*	46	7	14	2	0.29	0.31	1.00
Asr. alluaudi	*Ast. burtoni*	78	14	5	0	0.00	0.08	0.58
Asr. alluaudi	*Ast. calliptera*	72	14	8	2	0.14	0.10	0.66
Asr. alluaudi	*Ast. sparsidens*	61	13	14	1	0.08	0.27	0.45
Ast. burtoni	*Ast. calliptera*	81	14	5	0	0.00	0.07	0.59
Ast. burtoni	*Ast. sparsidens*	67	11	13	2	0.18	0.20	1.00
Ast. calliptera	*Ast. sparsidens*	69	12	13	3	0.25	0.18	0.70

Supplementary References

1 Haberyan, K. A. & Hecky, R. E. The Late Pleistocene and Holocene stratigraphy and paleolimnology of Lakes Kivu and Tanganyika. *Palaeogeo Paleoclim Paleoecol* **61**, 169-197 (1987).

2 Beadle, L. C. *The inland waters of tropical Africa : an introduction to tropical limnology*. 2nd edn (Longman, 1981).

3 Vernot, B. & Akey, J. M. Resurrecting surviving Neandertal lineages from modern human genomes. *Science* **343**, 1017-1021 (2014).

4 Terai, Y., Mayer, W. E., Klein, J., Tichy, H. & Okada, N. The effect of selection on a long wavelength-sensitive (*LWS*) opsin gene of Lake Victoria cichlid fishes. *Proc Natl Acad Sci U S A* **99**, 15501-15506 (2002).

5 Yokoyama, S. & Radlwimmer, B. The "five-sites" rule and the evolution of red and green color vision in mammals. *Mol Biol Evol* **15**, 560-567 (1998).

6 Terai, Y. *et al.* Divergent selection on opsins drives incipient speciation in Lake Victoria cichlids. *Plos Biol* **4**, 2244-2251 (2006).

7 Seehausen, O. *et al.* Speciation through sensory drive in cichlid fish. *Nature* **455**, 620-626 (2008).

8 Bryant, D., Bouckaert, R., Felsenstein, J., Rosenberg, N. A. & RoyChoudhury, A. Inferring species trees directly from biallelic genetic markers: Bypassing gene trees in a full coalescent analysis. *Mol Biol Evol* **29**, 1917-1932 (2012).

9 Bouckaert, R. *et al.* BEAST 2: a software platform for Bayesian evolutionary analysis. *PLoS Comput Biol* **10**, e1003537 (2014).

10 Ho, S. Y. *et al.* Time-dependent rates of molecular evolution. *Mol Ecol* **20**, 3087-3101 (2011).

11 Ho, S. Y. & Larson, G. Molecular clocks: when times are a-changin'. *Trends Genet* **22**, 79-83 (2006).

12 Genner, M. J. *et al.* Age of cichlids: New dates for ancient lake fish radiations. *Mol Biol Evol* **24**, 1269-1282 (2007).

13 Talbot, M. R. & Williams, M. A. in *The Nile* 37-60 (Springer, 2009).

14 Williams, M. A. & Talbot, M. R. in *The Nile* 61-72 (Springer, 2009).

15 Van Damme, D. & Pickford, M. The late Cenozoic Thiaridae (Mollusca, Gastropoda, Cerithioidea) of the Albertine Rift Valley (Uganda-Congo) and their bearing on the origin and evolution of the Tanganyikan thalassoid malacofauna. *Hydrobiologia* **498**, 1-83 (2003).

16 Scholz, C. A. *et al.* East African megadroughts between 135 and 75 thousand years ago and bearing on early-modern human origins. *Proc Natl Acad Sci USA* **104**, 16416-16421 (2007).

17 Danley, P. D. *et al.* The impact of the geologic history and paleoclimate on the diversification of East African cichlids. *Int J Evol Biol* **2012**, 574851 (2012).

18 Salzburger, W., Van Bocxlaer, B. & Cohen, A. S. Ecology and evolution of the African Great Lakes and their faunas. *Annu Rev Ecol Sys* **45**, 519-545 (2014).

19 Blome, M. W., Cohen, A. S., Tryon, C. A., Brooks, A. S. & Russell, J. The environmental context for the origins of modern human diversity: a synthesis of regional variability in African climate 150,000–30,000 years ago. *J Hum Evol* **62**, 563-592 (2012).

20 Cohen, A. S. *et al.* Ecological consequences of early Late Pleistocene megadroughts in tropical Africa. *Proc Natl Acad Sci* **104**, 16422-16427 (2007).

21 Johnson, T. C., Kelts, K. & Odada, E. The holocene history of Lake Victoria. *Ambio* **29**, 2-11 (2000).

22 Patterson, N. *et al.* Ancient admixture in human history. *Genetics* **192**, 1065-1093 (2012).

23 Miyagi, R. *et al.* Correlation between nuptial colors and visual sensitivities tuned by opsins leads to species richness in sympatric Lake Victoria cichlid fishes. *Mol Biol Evol* **29**, 3281-3296 (2012).

24 Martin, S. H., Davey, J. W. & Jiggins, C. D. Evaluating the use of ABBA–BABA statistics to locate introgressed loci. *Mol Biol Evol* **32**, 244-257 (2015).

25 Sturmbauer, C., Baric, S., Salzburger, W., Rüber, L. & Verheyen, E. Lake level fluctuations synchronize genetic divergences of cichlid fishes in African lakes. *Molecular Biology and Evolution* **18**, 144-154 (2001).

26 Koblmüller, S. *et al.* Age and spread of the haplochromine cichlid fishes in Africa. *Mol Phylogenet Evol* **49**, 153-169 (2008).

27 Friedman, M. *et al.* Molecular and fossil evidence place the origin of cichlid fishes long after Gondwanan rifting. *Proc R Soc B* **280**, 20131733 (2013).
28 Genner, M. J., Ngatunga, B. P., Mzighani, S., Smith, A. & Turner, G. F. Geographical ancestry of Lake Malawi's cichlid fish diversity. *Biol Lett* **11**, 20150232 (2015).
29 Eaton, D. A. R. & Ree, R. H. Inferring phylogeny and introgression using RADseq data: An example from flowering plants (Pedicularis: Orobanchaceae). *Syst Biol* **62**, 689-706 (2013).
